# Calycosin-loaded nanoliposomes as potential nanoplatforms for treatment of diabetic nephropathy through regulation of mitochondrial respiratory function

**DOI:** 10.1186/s12951-021-00917-1

**Published:** 2021-06-13

**Authors:** Chunrong Huang, Lian-Fang Xue, Bo Hu, Huan-Huan Liu, Si-Bo Huang, Suliman Khan, Yu Meng

**Affiliations:** 1 Department of Gastroenterology, The First Hospital Affiliated To Jinan University, Guangzhou, China; 2 Department of Clinical Pharmacy, The First Affiliated Hospital of Jinan University, Guangzhou, China; 3 Department of Nephrology, The First Hospital Affiliated To Jinan University, NO.613, Huangpu Avenue West, Guangzhou, 510150 China; 4 Department of Cerebrovascular Diseases, The Second Affiliated Hospital of Zhengzhou University, Zhengzhou, China; 5 Central laboratory, the Fifth Affiliated Hospital of Jinan University, Heyuan, China

**Keywords:** Diabetic nephropathy, ROS, Calycosin, Nanoliposome, Mitochondria

## Abstract

**Backgrounds:**

One of the most common complications in diabetic nephropathy is generation of high levels of ROS which can be regulated by herbal antioxidants. However, polyphenols like calycosin, the bioactive compound of *Radix astragali* suffer from low solubility and poor bioavailability.

**Methods:**

Therefore, in the present study, calycosin-loaded nanoliposomes were fabricated and characterized by TEM, DLS and FTIR techniques. Afterwards, the drug loading (DL) and entrapment efficiency (EE), drug release, solubility, stability, and pharmacodynamic assays were performed. Finally, the antinephropathic effects of calycosin-loaded-nanoliposomes on mitochondria of kidney cells were explored by MTT, ROS, MDA, mitochondrial respiratory function assays.

**Results:**

The result showed that the size, hydrodynamic radius, zeta potential, EE, and DL were, 80 nm, 133.99 ± 21.44 nm, − 20.53 ± 3.57, 88.37 ± 2.28%, and 7.48 ± 1.19%, respectively. The outcomes of in vitro release assay showed that calycosin-loaded nanoliposomes were significantly slow-release in dialysis media with pH 1.2, pH 6.9 and pH 7.4, at about 30 min, the dissolution of calycosin from nanoliposome became almost complete, and after 2 months, the calycosin-loaded nanoliposomes were still stable. Pharmacokinetic assay revealed that the AUC_0−t_ of calycosin in calycosin-loaded nanoliposome group was 927.39 ± 124.91 μg/L*h, which was 2.26 times than that of the free calycosin group (**P < 0.01). Additionally, the MRT_0−t_ and t_1/2_ of calycosin in the calycosin-loaded nanoliposome group were prolonged by 1.54 times and 1.33 times than that of free calycosin group, respectively (*P < 0.05). Finally, it was shown that calycosin-loaded nanoliposomes regulated the viability, ROS production, lipid peroxidation and function of mitochondria in kidney cells of diabetic rats as a model of diabetic nephropathy.

**Conclusion:**

In conclusion it may be suggested that new therapies based on nano-formulated calycosin can restore mitochondrial function which can improve diabetic nephropathy.

**Graphic abstract:**

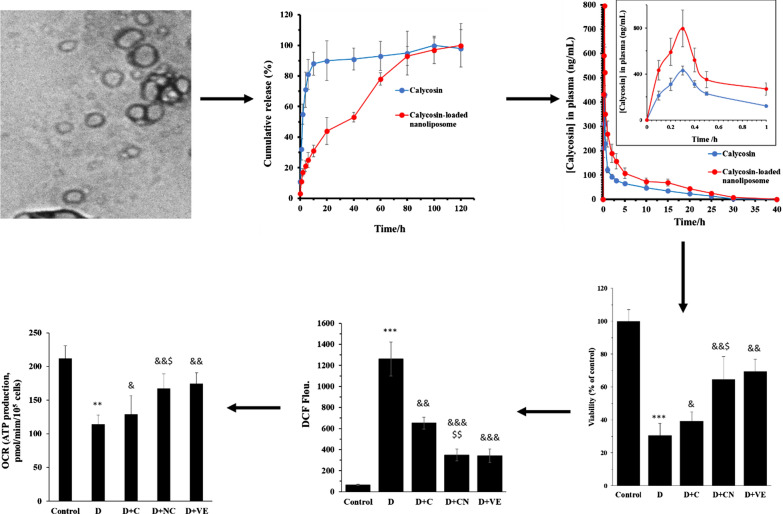

## Introduction

Diabetes is the most common metabolic disease due to a relative decrease in insulin secretion or function [1, 2]. Type 2 diabetes is the largest epidemic of the century and is currently the fastest growing disease among other disorders worldwide [3]. Complications of diabetes result in two groups of short-term disorders such as hyperglycemia, hyperuricemia, overeating and the appearance of glucose in the urine and long-term or chronic disorders including cardiac, renal, neurological, ocular and ocular injuries [4].

One of the most common microvascular complications in diabetes mellitus is nephropathy, which has been experienced by more than one patient with diabetes mellitus [5]. The kidneys play an important role in the filtration of blood waste products and are more vulnerable than other tissues in high blood sugar conditions [6]. On the other hand, diabetes mellitus is associated with oxidative stress, which is caused by an increase in free oxygen radicals, hydroxyl, or a decrease in the antioxidant defense system [7, 8]. Oxidative stress caused by high blood sugar in diabetes is also involved in the progression of diabetes complications, including nephropathy [8].

Hyperglycemia in diabetic patients leads to the production of more reactive oxygen species (ROS) in the body [9]. Oxidative stress and cell death that ultimately led to tissue damage in the kidney [9]. Mitochondria are the main source of ROS production in cells and ROS production through the mitochondrial respiratory chain is the main cause of hyperglycemia damage [10].

Today, with two strategies to control blood sugar and reduce oxidative stress in renal mitochondria, it is possible to reduce diabetic nephropathy due to oxidative stress. Treatment of hyperglycemia with chemical drugs or insulin causes several side effects such as chronic anorexia, cerebral atrophy and insulin-induced fatty liver [11]. Therefore, one of the most effective and economical ways to better control the effects of hyperglycemia is to neutralize the high levels of ROS produced and to use herbal antioxidants that are safe and tolerable. For example, it has been indicated that luteolin can show antidiabetic nephropathy in streptozotocin-stimulated diabetic rats [12], or mangiferin mitigates diabetic nephropathy by regulating oxidative stress-mediated signaling pathways [13]. Also, it has been indicated that ursolic acid provides antidiabetic nephropathy through downregulation of oxidative stress and expression of inflammatory mediators in vivo [14]. Some other bioactive compounds such as diosmin [15], ginger [16], and resveratrol [17] have also shown to inhibit the induction of diabetic nephropathy via regulation of oxidative stress and mitochondrial dysfunction.

One of the suggested treatments for the modulation of oxidative stress and mitochondrial function in diabetic nephropathy is the use of calycosin as a potent antioxidant [18].

Dry root extract of *Radix astragali* is extensively utilized for the mitigation of hypertension [19], inflammation [20], diabetes [21], and cancer [22]. The composition of its dry root extract contains several active metabolites, such as calycosin, saponins and several isoflavonoids [23]. Among these, calycosin provides the main activity of the dry root extract and is known as the typical bioactive compound of *Radix astragali* [23].

However, as calycosin as a polyphenol shows low solubility property which results in poor absorption and bioavailability and insufficient permeation, makes it difficult to be used as a potential candidate in the development of active drugs [24]. Therefore, some nano-based carriers can be designed to enhance solubility, bioavailability, efficacy, and drug delivery of drugs/compounds [25–28].

Nanoliposomes are colloidal structures consisting of a bilayer membrane [29]. In aqueous solutions, the hydrophobic groups of phospholipids orient inward and their hydrophilic groups orient outward [29]. Today, these nanostructures are used as carriers of drugs, genes, as well as water-insoluble compounds both in vitro and in vivo [30, 31]. The ability of these nanostructures to encapsulate large amounts of drugs, minimizing unwanted side effects, high efficacy and low toxicity has attracted the interest of researchers. Researchers have studied the delivery of different drugs by these nanocarriers [31]. The results have shown that by using nanoliposomes, drug delivery to target cells and the effectiveness of drugs are improved [30, 31]. For example, it has been indicated that the solubility and stability and potency of some plant-based compounds such as curcumin [32], fisetin [33], and resveratrol [34] can be improved after loading of these compounds into the nanoliposomes. In this paper, in addition to fabrication of calycosin-loaded nanoliposomes and their characterization, the protective effects against high glucose (HG)-induced oxidative stress in human kidney cells as a model of diabetic nephropathy were investigated.

## Materials and methods

### Materials

Calycoin, soybean phosphatidylcholine (SPC), cholesterol, 1,2-distearoyl-sn-glycero-3-phosphoethanolamine-N-[amino(polyethylene glycol)-2000] (DSPE-mPEG2000), 3-(4,5-dimethylthiazol-2-yl)-2,5-diphenyl-2H-tetrazolium bromide (MTT), and fetal bovine serum (FBS) were purchased from Sigma Co., Ltd. (Miami, USA). All other materials were of analytical grade.

### Preparation of calycosin-loaded nanoliposomes

Calycosin-loaded nanoliposomes were prepared through thin film evaporation-sonication method as described previously [35, 36]: SPC, cholesterol, DSPE-mPEG2000, and calycosin were dissolved with 12 mL methanol. The molar ratio of SPC to cholesterol and the mass ratio of the calycosin to the total lipid (SPC and cholesterol) (w/w) at the condition of 5% content of DSPE-mPEG2000 were 50∶38, 1:10, respectively.

The mixture was then dried (vacuum and 55 °C) to form a thin film in the rotary evaporation apparatus. Afterward, the samples were hydrated with 20 mL double distilled water, sonicated (70 w) for 15 min in an ice bath, extruded by filtering (0.45 μm and 0.22 μm), and finally freeze-dried at − 20 °C.

### Characterization of calycosin-loaded nanoliposomes

Scanning electron microscope (SEM, TESCAN vega3, Czech Republic) and transmission electron microscopy (TEM, FEI TECHNI, G2-USA) were used to analyze the morphology and size of calycosin-loaded nanoliposomes, respectively. Zeta Sizer (Malvern, UK) was employed to assess the particle size and charge distribution of calycosin-loaded nanoliposomes by dissolving them in water solution at room temperature. Fourier transform infrared spectroscopy (FTIR) within 4000–400 cm^−1^ range was used employing on PerkinElmer Spectrum FT-IR spectrometer (USA).

### Entrapment efficiency (EE), drug loading (DL), and in vitro drug release of calycosin-loaded nanoliposomes

The EE and DL of the prepared calycosin-loaded nanoliposomes were determined by the dialysis method described previously [35, 37]. Briefly, 700 μL of the prepared calycosin-loaded nanoliposomes solution was added into a dialysis bag (cutoff of 8000), followed by putting into 20 mL of dialysis medium (PBS, pH 7.4). The samples were then shacked (10 h), and centrifuged (3000 rpm, 2 min) to determine the free calycosin concentration in the supernatant. Also, 500 µL of the calycosin-loaded nanoliposomes were added by 1500 μL of methanol, vortexed (15 in), sonicated (5 min), centrifuged (3000 rpm, 2 min) to calculate the total calycosin concentration (*C*_0_). 20 mL of the prepared calycosin-loaded nanoliposomes powder (*V*_0_) was weighted to calculate the solid powder mass (*W*_0_). 25 µl of the sample was injected into a HPLC system containing Phenomenex ODS analytical column (150 mm × 4.6 mm, 5 μm) with a guard column (30 mm × 10 mm, 3 μm) at 40 °C. The mobile phase was a mixture of methanol: double distilled water as follows:0.05 M KH2PO4 = 40:60:5, pH 4.0, with a flow rate of 1.0 ml/min [37].

The percentage of EE and the DL were then determined according to the formula as follows [35, 37]:

$${\text{EE }}\left( \% \right) \, = \, \left( {{\text{C}}_{0} - {\text{C}}_{{1}} } \right)/{\text{C}}_{0} \times {1}00,$$

$${\text{DL }}\left( \% \right) \, = {\text{ C}}_{0} \cdot {\text{V}}_{0} \cdot {\text{EE}}/{\text{W}}_{0} \times {1}00.$$

The simulated gastric fluid was 0.1 mol/L HCl (100 mM, pH 1.2) supplemented with 0.5% Tween-80, the simulated intestinal fluid was PBS buffer (25 mM, pH 6.9) containing 0.5% Tween-80, and the simulated physiological buffer was PBS (150 mM, pH 7.4) containing 0.5% Tween-80. The cumulative drug release was then determined using the following equation [35]:

$${\text{Cumulative release rate }}\left( \% \right) \, = \, [{\text{V}}_{{1}} \times \, ({\text{C}}_{{1}} \cdots + {\text{C}}_{{{\text{i1}}}} ) \, + {\text{V}}_{{2}} \times {\text{C}}_{{\text{i}}} ]/ \, \left( {{\text{V}}_{{\text{o}}} \times {\text{C}}_{{\text{o}}} } \right) \, \times {1}00$$

where, *V*_1_ is the sampling amount, *V*_2_ is the volume of the dialysis medium, *C*_1_–*C*_*i*_ is the concentration of calycosin at each time point, and *V*_0 _and *C*_0_ are the volume and concentration of calycosin-loaded nanoliposomes added to the dialysis bag. Stability of calycosin-loaded nanoliposomes was explored for about two months at 37 °C by using particle size and encapsulation efficiency of the reconstituted nanoliposome.

### Dissolution studies

The dissolution studies were performed based on the previous study [37]. Briefly, the dissolution medium (pH 7.4, PBS, 900 ml) under stirring (100 rpm) was added by calycosin-loaded nanoliposomes which is equivalent to 50 mg of drug and at different time intervals, 15 mL samples were removed, replaced, filtrated (0.45 m), and dissolved in mixed solvent (methanol: distilled water = 5.5:3.5, v/v). Finally, 25 µl of the sample was injected into a HPLC system. The rest of the setup was similar to the above-mentioned explanations.

### Pharmacokinetics of calycosin-loaded nanoliposomes in rats

Ten male Sprague–Dawley rats were randomly divided into three groups (*n* = 8) as control, treated with calycosin, and treated with calycosin-loaded nanoliposomes (dissolved in 1% ethanol). The doses of calycoin groups were 30 mg/kg for oral administration. After the rats were intragastrically administered with free or Nano-formulated calycosin, the 0.5 mL blood was taken from the fundus venous plexus of each rat at different time intervals. The blood samples were then centrifuged (4000 rpm, 10 min), and the plasma was removed and to stored − 80 °C. The content of the drug in the plasma sample was then calculated by the established LC-MS/MS method based on the previous report [35].

### Animal handling

To adapt to the environment, two weeks before the experiments, the Sprague–Dawley rats (male, 230–250 g) were placed in separate standard cages and light cycles for 12 h of light and 12 h of darkness and access to sufficient water and food at 22 °C.

### Diabetes induction method

Diabetes was induced in mice by injecting streptozotocin purchased from Sigma (USA). Streptozotocin was dissolved in a citrate buffer (pH 6.5, 0.1 M) and injected at a dose of 65 mg/kg intraperitoneally. Blood glucose was measured before the test and one week after streptozotocin injection, and mice with blood glucose above 200 dl/mg were considered diabetic. The calycosin or calycosin-loaded nanoliposomes were dissolved in PBS until it was ready to be injected, and the mice received 30 kg/mg of calycosin-loaded nanoliposomes for 4 weeks after becoming diabetic. Vitamin E (100 mg/kg) was also used as a positive control group.

### Animal groups and isolation of mitochondria from kidney

Animals were randomly divided in 5 groups which each group consisted of 6 male mice. Group 1: control (receiving no medication), group 2: diabetic group receiving streptozotocin at a dose of 65 mg / kg intraperitoneally, group 3: received streptozotocin at 65 mg/kg and after confirmation of diabetes, they received calycosin at 60 mg / kg intraperitoneally in 4 weeks, group 5: received calycosin-loaded nanoliposomes at 60 mg / kg intraperitoneally in 4 weeks, group 5: The positive control group was diabetic mice that received 100 mg /kg of vitamin E for 4 weeks. After the treatment period, the animals were anesthetized with ether, then the kidney tissue was removed, washed with mannitol buffer, and homogenized with normal PBS by homogenizer. The homogenized tissue is first centrifuged (10,000 rpm, 10 min) and the supernatant was then discarded and the sediment containing mitochondria was dispersed in Tris buffer on ice [38].

### Determination of protein concentration

Protein concentration was measured as standard using bovine serum albumin (BSA). In all experiments, mitochondria with a protein concentration of 1 mg/ml were used.

### MTT assay

MTT assay was done to assess the mitochondria viability. 100 µl of mitochondrial supernatants were mixed by MTT stock solution (5 mg mL^−1^) for 4 h, followed by removing the supernatants, addition of 150 μL DMSO for 5 min, and reading the absorbance of the samples at 570 nm using a microplate reader (BIO-RAD microplate reader-550).

### Measurement of intracellular reactive oxygen species (ROS)

The generation of intracellular ROS was investigated through the dichlorodihydrofluorescein diacetate (DCFH-DA) assay. Briefly, 20 µl of mitochondrial supernatants were incubated with a DCFH-DA probe (10 μM) for 30 min. Then, the fluorescence intensity of the samples was read at λ_ex_/λ_em_ of 485 nm/530 nm using a spectrofluorometer (CLARIOstar, Offenburg, Germany).

### Measurement of lipid peroxidation

The level of end product of lipid peroxidation (malondialdehyde [MDA] was measured through thiobarbituric acid reactive substances (TBARS) analysis [39]. Briefly, 20 µl of mitochondrial supernatants were added by 390 µL trichloroacetate (28%) and were centrifuged (3500 g for 15 min). Afterward, the supernatant was mixed with TBA (0.2% in sodium sulfate) on boiling water and incubated for 40 min, followed by the addition of n-butanol. Finally, the absorbance of samples was read at 532 nm using a microplate reader (BIO-RAD microplate reader-550).

### Measurement of oxygen consumption rate (OCR)

The OCR of cells was quantified employing a Seahorse XFe24 Analyzer (Agilent, MA, USA). Briefly, after treatment, the cells were washed, incubated with XF DMEM and D-glucose, and incubated in a non-CO_2_ incubator for 30 min at 37 °C. The sequential additions of oligomycin with a concentration of 1 μM (ATP synthase inhibitor), FCCP with a concentration of 2 μM (mitochondrial oxidative uncoupler), and rotenone with a concentration of 1 μM (mitochondrial electron transport chain complex I inhibitor) was done to assess the ATP production, maximal respiration, and spare respiratory capacity employing Seahorse Wave software (http://www.seahorsebio.com). The membrane potential was also determined using Cellular Membrane Potential Assay Kit (Fluorometric-Red) (ab176765) based on manufacturer's instructions provided.

### Statistical analysis

Statistical analysis was carried out using Student’s *t*-test and one-way analyses of variance (ANOVA). Results were reported as means ± standard errors (SEs) of three (*n* = 3) independent experiments. *P* < 0.05 was considered significant.

## Results and discussion

### Characterization of calycosin-loaded nanoliposome

**Fig. 1:**
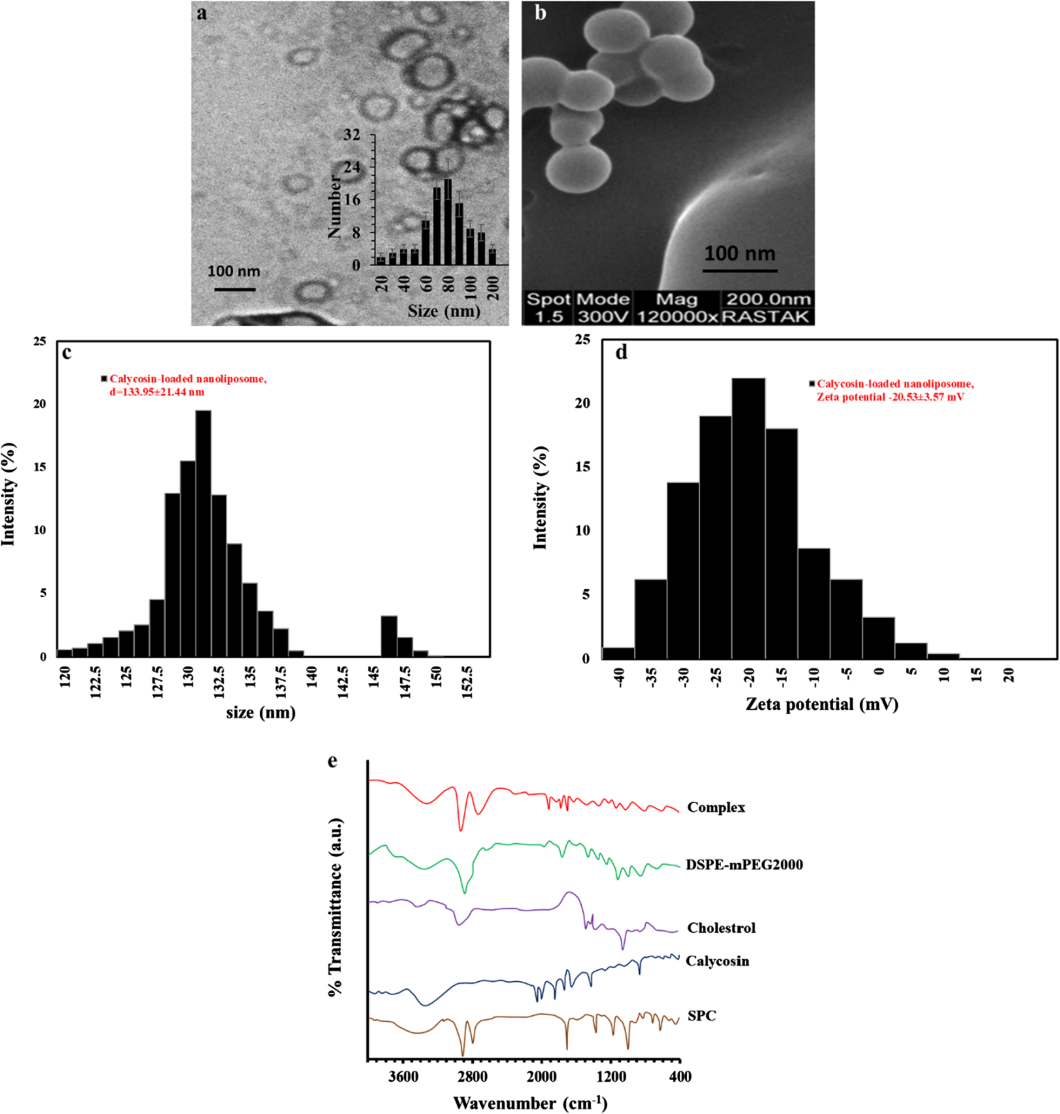
**a** TEM (inset: size distribution histogram), **b** SEM, **c** DLS histogram, **d** zeta potential histogram, **e** FTIR analysis of fabricated calycosin-loaded nanoliposomes. The experiment was done at room temperature

The size of calycosin-loaded nanoliposomes was characterized by TEM analysis (Fig. 1a). The histogram of size distribution determined by TEM revealed the synthesized drug-loaded nanoliposomes showed a size range of 20–200 nm with average size of around 80 nm (Fig. 1a, inset, 100 nanoliposomes were counted in the analysis). The SEM image showed that synthesized calycosin-loaded nanoliposomes were spherical NPs with very homogeneous distribution and a size of around 80 nm (Fig. 1b), which is in good agreement with TEM analysis. DLS is known as one of the most sensitive approaches employed for the determination of the colloidal stability of nano-based materials based on the hydrodynamic radius [40]. The calycosin-loaded nanoliposomes were also characterized by DLS technique and the result showed that the average particle size distribution was in the range of 120–150 nm with a mean size of 133.99 ± 21.44 nm (PDI = 0.201 ± 0.068) (Fig. 1c). The narrow distribution of histogram also indicates the uniform distribution of calycosin-loaded nanoliposomes.

Zeta potential study was also done to measure the charge distribution of fabricated nanoliposomes. It was shown that the mean zeta potential of calycosin-loaded nanoliposomes was -20.53 ± 3.57 mV (Fig. 1d), indicating the good colloidal stability of nanoparticles.

Calycosin is a typical polyphenol with poor hydrophilic features. The partial interaction of the calycosin with the polar part of the phospholipid can result in increasing their dissolution rate. It has been indicated that calycosin can interact with liposome membranes [41]. According to the TEM and SEM images, the structure of drug-loaded nanoliposome was obvious, and the loading of calycosin did not destabilize the typical structure of lipids.

The formation of the of calycosin-loaded nanoliposomes complex was assessed by FTIR spectroscopy by comparing the vibration band of the complex with the individual compounds. As shown in Fig. 1e, there were some differences in different regions between the complex and each of its individual compounds. The spectrum of complex revealed a new peak at 1331 cm^−1^, which was not appeared in other components. This spectrum also demonstrated a reduced intensity in the peak at 1697 cm^−1^. These data indicated that hydrogen-bonding between calycosin and SPC may play an important role during the final complex as calycosin-loaded nanoliposomes. These differences were determined at N–O band vibration regions (1400–1300 cm^−1^) and P-O band vibration regions (1700–1800 cm^−1^). Therefore, it can be indicated that hydrogen-bonding forms between polar region of calycosin and cholin group of SPC.

### Drug loading and in vitro drug release

**Fig. 2:**
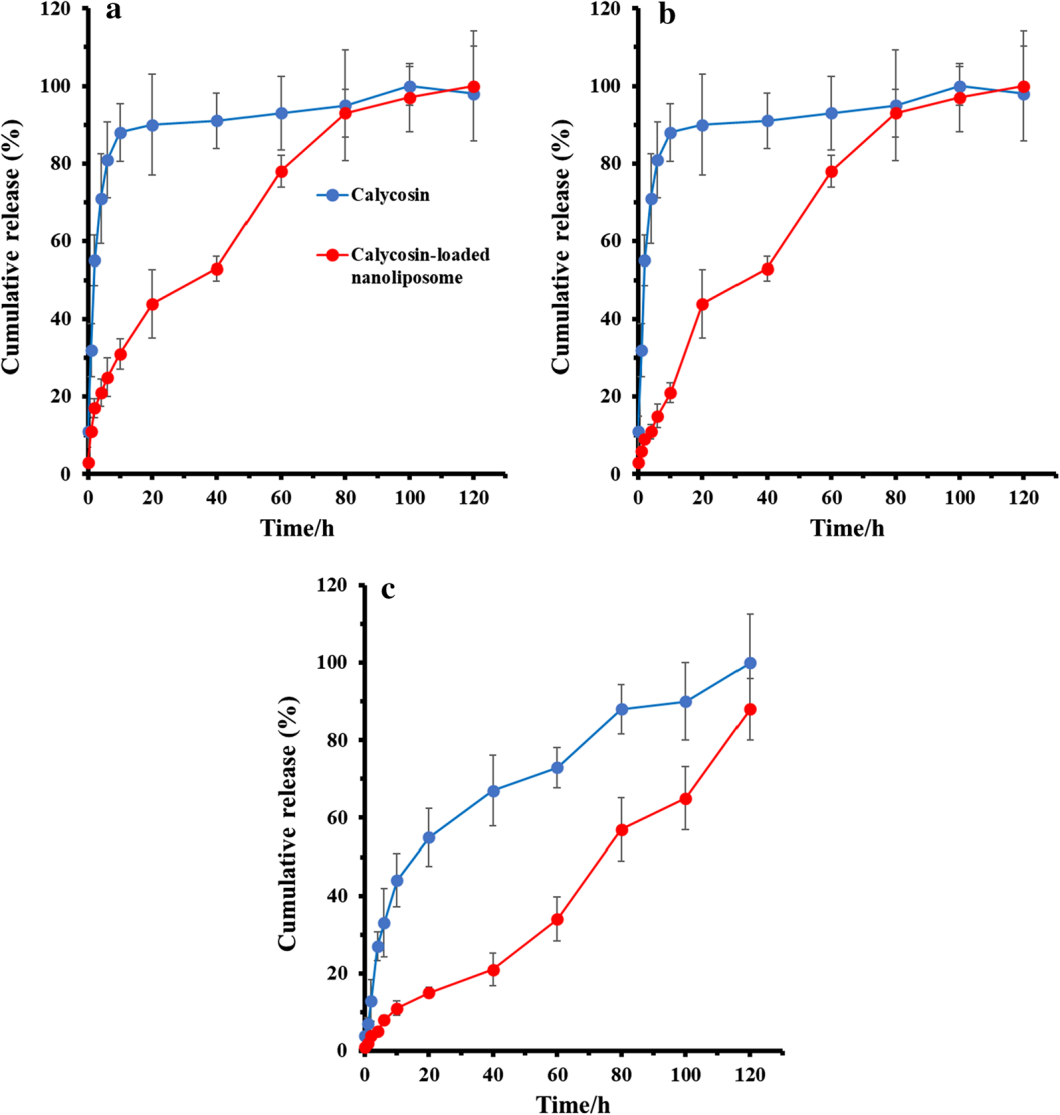
In vitro release of calycosin-loaded nanoliposome and free calycosin at different release media: **a** (pH 1.2), **b** PBS (pH 6.9), **c** PBS (pH 7.4) (mean ± SD, *n* = 3)

Based on the experimental set up mentioned in the method section, the EE was 88.37 ± 2.28%, and the DL was 7.48 ± 1.19%. The outcomes of in vitro release experiments were displayed in Fig. 2. In the simulated gastric medium, the cumulative release (%) of calycosin was about 32% at 1 h and almost complete release (%) at 20 h; calycosin-loaded nanoliposomes released 11% at 1 h, 44% at 20 h, and 100% at 80 h. In the simulated intestinal fluid, the cumulative release (%) of calycosin was about 32% at 1 h, 90% at 20 h, and almost complete release at 100 h; calycosin-loaded nanoliposome released 6% at 1 h, 44% at 20 h, and 100% at 120 h. In the simulated physiological fluid, the cumulative release (%) of calycosin was about 7% at 1 h, 55% at 20 h, and almost complete release at 120 h; calycosin-loaded nanoliposome released 2% at 1 h, 15% at 20 h, and 100% at > 120 h.

The outcomes of in vitro release assays indicated that calycosin-loaded nanoliposomes were remarkably slow-release in dialysis media with pH 1.2, pH 6.9 and pH 7.4, and the release rate in buffer solution with pH 1.2 was faster than that in buffer solutions with pH 6.9 and pH 7.4. This may be due to the reason that nanoliposome are vulnerable in acidic media and provide poor stability.

### Dissolution study

**Fig. 3:**
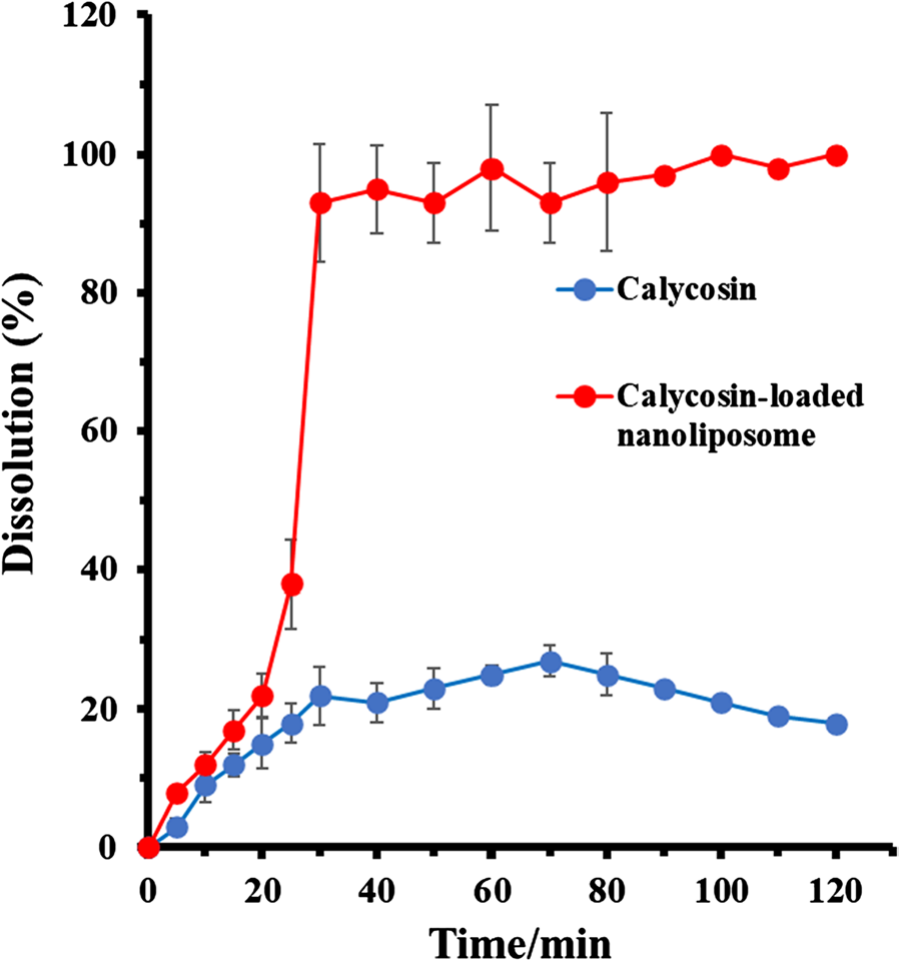
Dissolution behaviors of calycosin-loaded nanoliposome in PBS buffer (pH 7.4) (mean ± SD, *n* = 3)

Figure 3 displays the dissolution profile of calycosin from nanoliposome in PBS buffer (pH 7.4). At about 30 min, the dissolution of calycosin from nanoliposome became almost complete. It can be indicated that the nanoliposomes formed an encapsulation medium on contact with the solution and calycosin were entrapped in liposomes. However, for free drug, the dissolution rate of calycosin was low.

### Stability of calycosin-loaded nanoliposome

After 2 months, the appearance of calycosin-loaded nanoliposome still was uniform, and the prepared nanoliposome could form a nanoliposome dispersion upon contact with solution. Table 1 tabulated that particle size (nm) and EE (%) of calycosin-loaded nanoliposome have no significant change at 37 °C for 2 months.

**Table 1:** Stability of calycosin-loaded nanoliposome at the temperature of 37 °C for 2 months

Time (month, *n* = 3)	Average size (nm, *n* = 3)	Entrapment efficiency (nm, *n* = 3)
0	133.99 ± 21.44	88.37 ± 2.28%
1	149.07 ± 18.64	86.19 ± 1.37
2	163.71 ± 27.68	85.67 ± 1.59

### In vivo pharmacokinetics

**Fig. 4:**
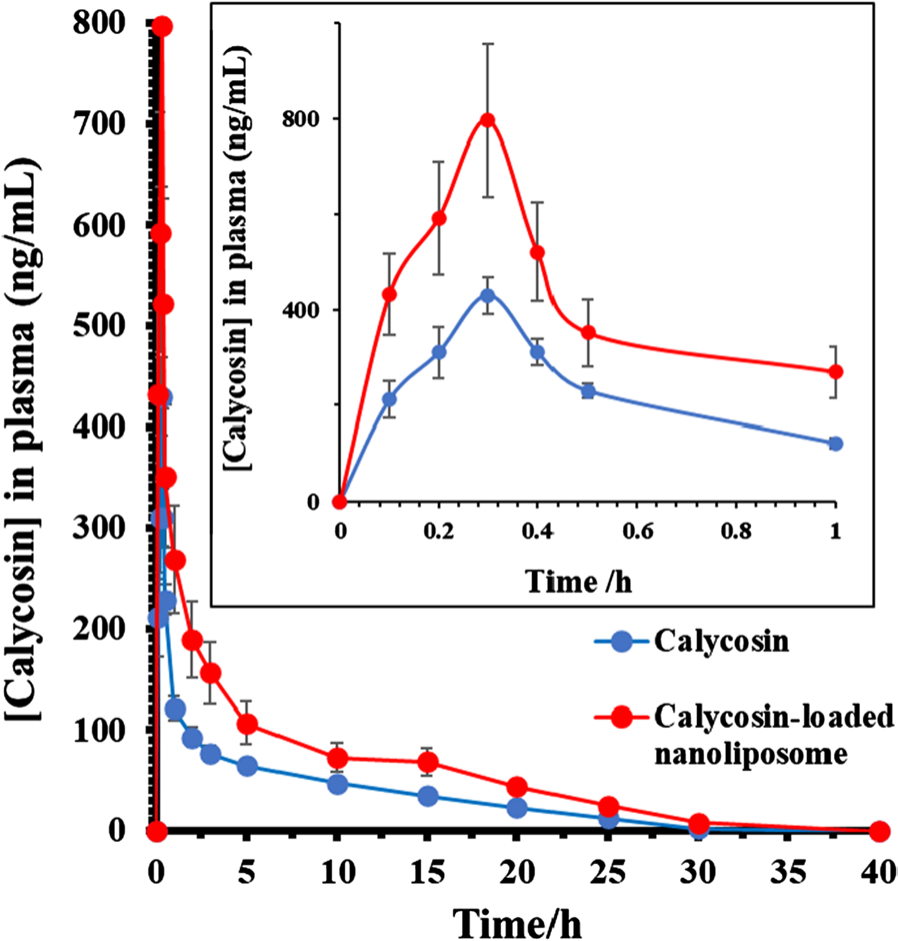
Pharmacokinetic curves of calycosin-loaded nanoliposome and free calycosin after oral administration of a single dose of calycosin (30 mg/kg) (mean ± SD, *n* = 5)

The pharmacokinetic curves of calycosin after oral administration of a single dose of calycosin and calycosin-loaded nanoliposome were demonstrated in Fig. 4. The outcomes indicated that under a single dose of calycosin (30 mg/kg), the plasma concentration of calycosin in the nanoliposome group was always higher than that in the free calycosin group. Also, the main pharmacokinetic parameters of calycosin in both groups were summarized in Table 2.

**Table 2:** Main pharmacokinetic parameters of free calycosin and calycosin-loaded nanoliposome group after oral administration (mean ± SD, *n* = 3)

Parameters	Unit	Free calycosin group	Calycosin-loaded nanoliposome group
AUC_0−*t*_	μg/L*h	409.11 ± 67.29	927.39 ± 124.91**
AUC_0−*∞*_	μg/L*h	421.09 ± 73.27	945.37 ± 158.91**
MRT_0−*t*_	h	8.68 ± 1.27	13.39 ± 1.93*
*t* _1/2_	h	6.98 ± 0.96	9.29 ± 0.88*
*T* _max_	h	0.19 ± 0.017	0.14 ± 0.019*
*C* _max_	μg/L*h	298.27 ± 35.81	331.52 ± 47.08

*P < 0.05, **P < 0.01 vs free calycosin group

*AUC* Area under the time-concentration curve, *t*_*1/2*_ Elimination half-life, *MRT* Mean residence time, *T*_*max*_ The time of peak concentration, *C*_*max*_ Peak concentration

The data revealed that under the iso-dose calycosin in both free calycosin- and calycosin-loaded nanoliposome-treated groups, the AUC_0−t_ of calycosin in calycosin-loaded nanoliposome group was 927.39 ± 124.91 μg/L*h, which was 2.26 times than that of the free calycosin group (**P < 0.01). Additionally, the MRT_0−t_ and t_1/2_ of calycosin in the calycosin-loaded nanoliposome group were prolonged by 1.54 times and 1.33 times than that of the free calycosin group, respectively (*P < 0.05). The data revealed that calycosin-loaded nanoliposome could not only mitigate the first-pass influence of calycosin to improve its oral absorption, but also prolong its resident time to provide the slow-release impact.

The pharmacokinetic data showed that calycosin-loaded nanoliposome could not only decrease the first-pass impact of calycosin to improve its oral absorption, but also prolong its mean bioavailability to provide the slow-release effect.

### MTT assay

**Fig. 5:**
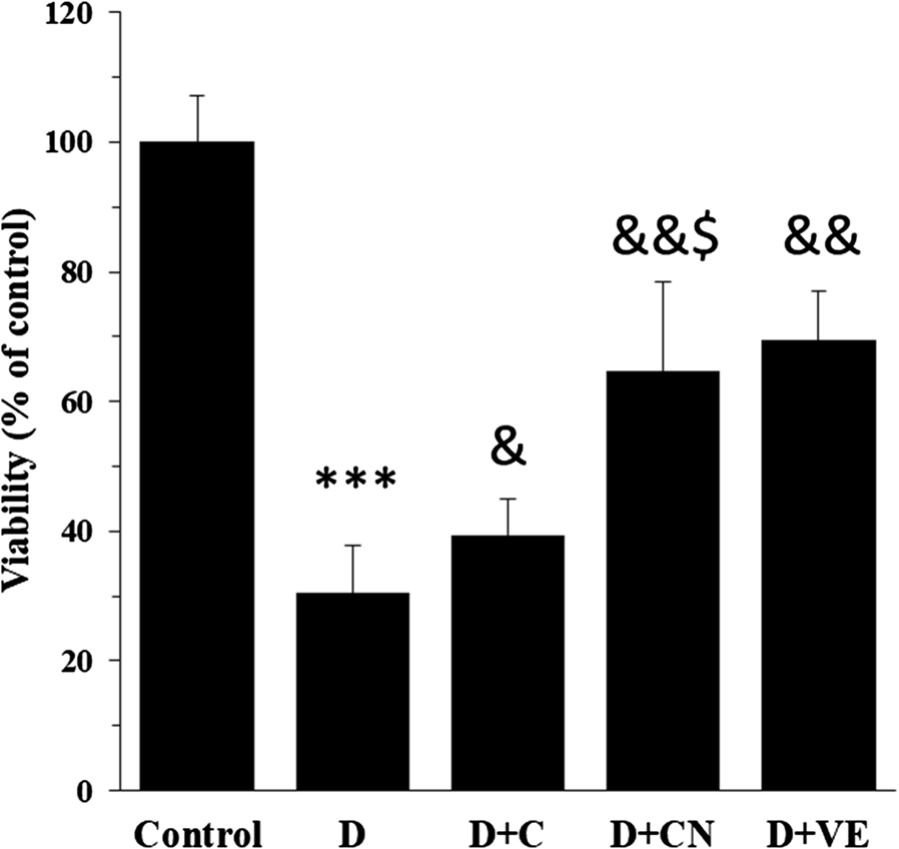
Protective effect of calycosin-loaded nanoliposome on mitochondria of diabetic rats. Data represent mean ± SEM. of three independent runs. ***P < 0.001 relative to control; ^&^*P* < 0.05, ^&^*P* < 0.01 relative to D-treated group; ^$^*P* < 0.05 relative to D + C- treated group. Diabetic group (D), diabetic group treated with calycosin (D + C), diabetic group treated with calycosin-loaded nanoliposome (D + CN), diabetic group treated with vitamin E (D + VE)

MTT assay was performed to assess the mitochondrial viability of diabetic rats treated with free calycosin or calycosin-loaded nanoliposomes. As shown in Fig. 5, the mitochondrial viability significantly decreased in diabetic rats (***P < 0.001) compared to the control group. However, treatment of the cells with calycosin and calycosin-loaded nanoliposome led to a significant recovery of mitochondrial viability in comparison with diabetic rats. Also, it was determined that the protective impact of calycosin-loaded nanoliposome was more significant (^$^*P* < 0.05) than free calycosin and was comparable with vitamin E (Fig. 3b). This outcome displayed that calycosin-loaded nanoliposome can be a potential candidate in the protection of the kidney cells against diabetes.

### ROS and MDA assay

**Fig. 6:**
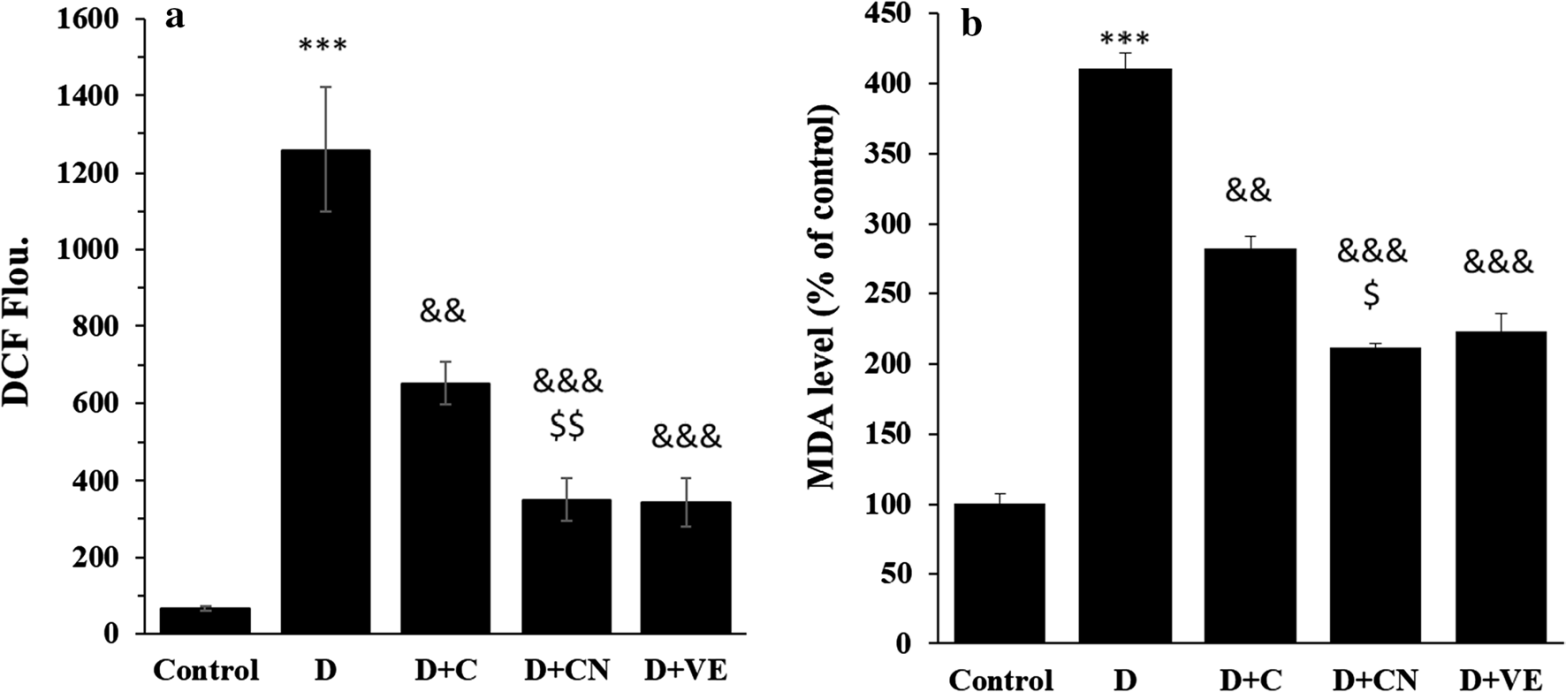
**A** ROS, **B** MDA levels in mitochondria of diabetic rats. Data represent mean ± SEM. of three independent runs. ***P < 0.001 relative to control; ^&&^*P* < 0.01, ^&&&^*P* < 0.001 relative to D-treated group; ^$$^*P* < 0.01 relative to D + C- treated group. Diabetic group (D), diabetic group treated with calycosin (D + C), diabetic group treated with calycosin-loaded nanoliposome (D + CN), diabetic group treated with vitamin E (D + VE)

The generation of ROS was assessed in diabetic rats through DCFH-DA assay. As depicted in Fig. 6a, diabetic rats showed a significant (***P < 0.001) increase in DCF fluorescence intensity associated with the ROS level. However, treatment with free calycosin and calycosin-loaded nanoliposome potentially reduced the generation of ROS, as indicated by the lower DCF fluorescence intensity (Fig. 6a). MDA, which is the end product of lipoperoxidation and as a potential indicator of oxidative stress was assessed to examine the protective effect of calycosin-loaded nanoliposome against oxidative stress in diabetic rats. A significant increase (***P < 0.001) in the MDA level was detected in diabetic rats relative to the control cells, whereas treatment with calycosin or calycosin-loaded nanoliposome determined a remarkable decrease in lipid peroxidation (Fig. 6b). The results also indicated that the protective effect of calycosin-loaded nanoliposome against diabetes-induced generation of ROS (^$$^*P* < 0.01) and MDA (^$^*P* < 0.05) was more pronounced than free calycosin and was comparable with vitamin E. This data determined that treatment of diabetic rats with calycosin-loaded nanoliposome inhibited the increase in ROS and MDA level, mitigated lipoperoxidation of the cell membrane and decreased cell damage.

### Mitochondrial respiration dysfunction

**Fig. 7:**
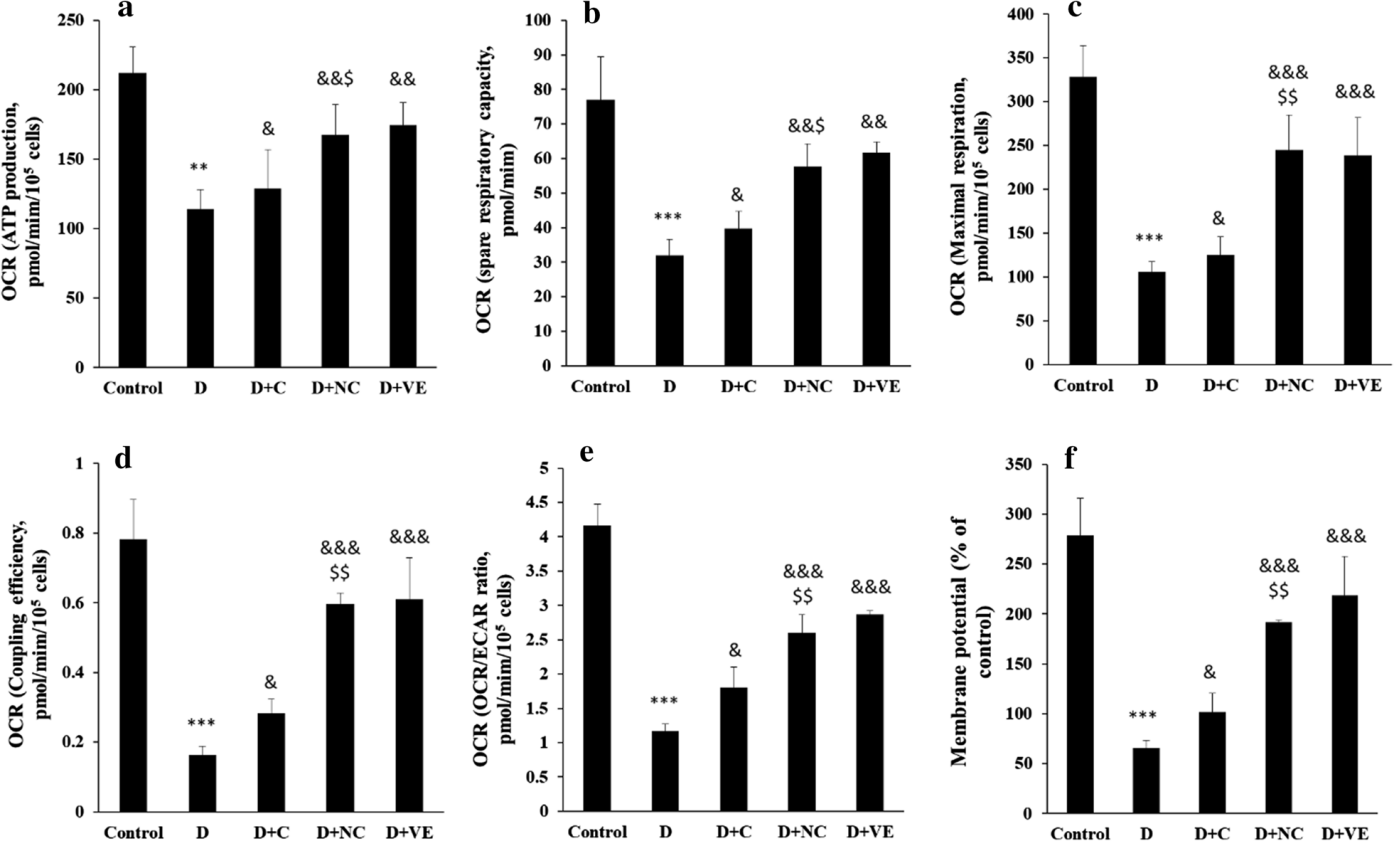
Calycosin-loaded nanoliposomes ameliorate mitochondrial dysfunction in diabetic rats. Data represent mean ± SEM. of three independent runs. **P < 0.01, ***P < 0.001 relative to control; ^&^*P* < 0.05, ^&&^*P* < 0.01, ^&&&^*P* < 0.001 relative to D-treated group; ^$^*P* < 0.05, ^$$^*P* < 0.01 relative to D + C- treated group. Diabetic group (D), diabetic group treated with calycosin (D + C), diabetic group treated with calycosin-loaded nanoliposome (D + CN), diabetic group treated with vitamin E (D + VE)

It has been well-documented that the mitochondrial function is important to improve cell survival [42]. To further assay the protective impact of calycosin-loaded nanoliposome on mitochondrial respiratory function, the OCR amounts and mitochondrial membrane potential of diabetic rats treated with calycosin or calycosin-loaded nanoliposome were determined. Indeed, in this assay after the sequential addition of some mitochondrial stressors, OCR values of the mitochondria were analyzed using a Seahorse XFe24 Analyzer (Fig. 7a–f). It was seen that diabetic rats showed a significant decrease in ATP production (Fig. 7a), spare respiratory capacity (Fig. 7b), maximal respiration (Fig. 7c), coupling efficiency (Fig. 7d), OCR/extracellular acidification rate (ECAR) ratio (Fig. 7e), and membrane potential (Fig. 7f) compared with those in the control group. However, calycosin-loaded nanoliposome determined to reverse these mitochondrial malfunctions with statistical significance relative to the free calycosin. Taken together, these data suggested that calycosin-loaded nanoliposome treatment could protect diabetic rats against oxidative stress-triggered mitochondrial respiration dysfunction.

Lack of strong antioxidant defense can lead to activation of the signaling pathway associated with oxidative stress in diabetic nephropathy [43, 44]. In fact, one of the most important cellular responses to high glucose concentrations is oxidative stress and increased production of ROS in the mitochondria and ultimately the occurrence of apoptosis in cells and tissues [45]. Physiologically, some endogenous antioxidant molecules such as superoxide dismutase, glutathione peroxidase, catalase, and bilirubin reductase are excreted by oxidative stress-induced renal cell damage [46]. But in people with diabetes the generation of free radical increases due to decrease in the level of antioxidant systems, resulting in a continuous chain reaction of lipid peroxidation mitochondrial dysfunction [46].

Due to the low solubility and poor bioavailability of polyphenols, therapeutic perspectives of bioactive polyphenols through their nanoformulations against diabetic neuropathy have been suggested [47]. Indeed, it has been documented that polyphenol-stabilized nanoparticles can show potential anti-diabetic activity [24, 48, 49].

The physicochemical stability of calycosin is influenced by different parameters and therefore their metabolism, bioavailability, and pharmacokinetic activities are changed. These drawbacks can be overcome by encapsulation of the calycosin into nano-based platforms. An increase in the bioavailability of calycosin-loaded nanoliposomes was observed with an enhancement in their solubility, sustained drug release, improvement of its pharmacokinetic features, and promotion of its antidiabetic-nephropathy effects. The antidiabetic-nephropathy activity of calycosin was reported to be increased after nanoliposome encapsulation.

In this study, evidence was provided that mitochondrial function was reduced in the predominant form of diabetic nephropathy in rats which is in good agreement with other reported studies [50–52]. We found that calycosin-loaded nanoliposomes play a key role in recovering mitochondrial function of kidney cells. This means that new therapies based on nano-formulated calycosin can restore mitochondrial function to normal and their content can improve or even stop chronic kidney disease.

## Conclusion

According to the results of the other studies and the data of the present study, it can be boldly claimed that calycosin can play a key role in reducing the damage caused by oxidative stress in kidney cells as an antioxidant. However, further and more extensive studies on the exact mechanism or molecular and cellular mechanisms influencing the pharmacological function of calycosin are necessary for its treatment or control of diabetic nephropathy and other diseases. The results of this study show that calycosin-loaded nanoliposomes can be useful in inhibiting the diabetes mellitus-induced kidney cell damage in which the cells are under oxidative stress due to recovery of mitochondrial function.

## Data Availability

The datasets used and analyzed during the current study are available from the corresponding author on reasonable request.
